# Clinical spectrum and diagnostic outcomes of patients with suspected inflammatory rheumatic disease in the emergency department: a retrospective study

**DOI:** 10.1007/s00296-026-06113-4

**Published:** 2026-06-05

**Authors:** Pavels Klimicevs, Frederic Christian Feindt, Phillip Kremer, Marie-Therese Holzer, Isabell Haase, Martin Krusche, Ina Kötter, Simon Melderis

**Affiliations:** 1 III. Department of Medicine, University Medical Center Hamburg-Eppendorf, Hamburg, Germany; 2 Hamburg Center for Kidney Health (HCKH), University Medical Center Hamburg-Eppendorf, Hamburg, Germany; 3 Clinic for Rheumatology and Immunology, Auenlandklinik, Bad Bramstedt, Germany

**Keywords:** Emergency Service, Hospital, Referral and Consultation, Rheumatic Disease, Decision Support Techniques, Health Resources, Arthritis, Rheumatoid, Polymyalgia Rheumatica, Reactive Arthritis

## Abstract

Little is known about patients presenting to the emergency department (ED) with rheumatological complaints. We aimed to characterize rheumatological patients in the ED and analyze the role of the rheumatologist in their management. This retrospective single-center cohort study analyzed 340 patients presenting to the emergency department (ED) between 2019 and 2023 with a suspected new inflammatory rheumatic disease (IRD). Clinical and process data were evaluated. In addition, multivariable logistic regression and a neural network model were employed to identify factors that are predictive of a subsequent diagnosis of IRD. Arthritis and arthralgia (55%) were the most common symptoms among patients with susIRD, but a wide spectrum of symptoms were observed, including myalgias (23%) and B-Symptoms (20%). Most patients presented as urgencies rather than true emergencies. Symptom duration prior to ED presentation was longer than 7 days in 69% of patients. However, 63% required in-patient management. Overall,y 62% of patients with susIRD were eventually diagnosed with an IRD. Immunosuppressive or anti-inflammatory medication was started in 92% of these. The most common diagnoses were polymyalgia rheumatica (7.9%), reactive arthritis/para infectious arthritis (7.3%) and rheumatoid arthritis (6.3%). Infections (10%) constituted the most common non-rheumatological diagnosis. A logistic regression model and a neural network could predict the presence of an IRD with accuracies of 74% and 60% respectively. Polyarthritis/Polyarthralgia and swollen joints were the strongest positive predictors for IRD. When consulting rheumatologists suspected an IRD, this impression was confirmed by the final diagnosis in 87% of patients. Integrating the rheumatologist’s impression into the models increased the accuracy of the logistic regression to 85% and the neural network to 77%. Most rheumatological patients present to the ED with arthritis/arthralgias and urgent rather than life-threatening conditions. Whilst our models could predict IRD with moderate accuracy, consulting rheumatologists demonstrated high diagnostic precision, indicating the need for rheumatological expertise in the ED.

**Supplementary Information:**

The online version contains supplementary material available at 10.1007/s00296-026-06113-4.

## Introduction

As healthcare workforce shortages increase, access to routine medical care becomes increasingly limited [1]. Consequently, patients are more frequently seeking care in the emergency department (ED).

Inflammatory rheumatic diseases (IRD) rarely result in life-threatening emergencies [2, 3]. However, data from the late 1980s showed that up to 8% of patients presenting to EDs could be classified as having a rheumatic condition [4].

A study that looked at patients with known IRD and their utilization of the ED found most patients to be relatively stable with a majority being classified as ≥ 4 on the Manchester Triage System (MTS) [5], indicating urgencies rather than true emergencies [2]. Fever, musculoskeletal pain and abdominal pain were the most common symptoms.

While there is some data on why patients with established IRD present to the ED; there is even less information on patients with rheumatological complaints but without a known IRD [6]. Most of this data focusses solely on patients with musculoskeletal symptoms rather than systemic inflammatory presentations [3, 7–11].

The symptoms leading to ED presentation and the diagnoses ultimately established remain poorly understood. In 1996 E.C. Smith identified the need for an acute rheumatology referral system for treatment centers and general practitioners/family doctors (GP), demonstrating that such a service is beneficial for patients and cost effective [12]. For some IRD (e.g. gout) we know that a rheumatological consultation (RC) of hospitalized patients significantly improves the diagnostic accuracy and adherence to established guidelines for short- and long-term treatment [13]. A study about referral to outpatient rheumatology for suspected rheumatic disease showed that a short telephone-based triage by a rheumatologist could correctly differentiate between inflammatory and non-inflammatory rheumatic diseases [14].

The exact set-up of EDs and the provision of healthcare to patients with suspected rheumatological emergencies varies around the world. Nevertheless, in the context of patients with potential IRD in the ED, the role of rheumatologists and rheumatological expertise in general remains poorly studied. One way to assess rheumatological expertise would be diagnostic accuracy. Machine learning algorithms and clinical decision support systems have made significant progress in recent years. They have been studied for the diagnosis of secondary hemophagocytic lymphohistiocytosis, diagnosing chronic pediatric rheumatic conditions and triaging musculoskeletal conditions [15–17].

This study aimed to investigate rheumatological presentations in the ED by addressing three key questions: (1) what is the spectrum of patients presenting with suspected inflammatory rheumatic disease (susIRD); (2) how accurately can IRD be distinguished from alternative diagnoses; and (3) what is the role and impact of rheumatological expertise in the ED?

## Methods

### Study design and setting

We performed a retrospective study of patients with rheumatological complaints that presented to the ED of the University Medical Center Hamburg-Eppendorf (UKE) between 2019 and 2023. The UKE is an academic tertiary care center with departments for all medical specialties. No rheumatologist is permanently staffed at the ED. When rheumatological expertise is needed, a rheumatological consultation (RC) is requested by the ED-team and patients are then reviewed by a rheumatologist.

### Participants

We screened electronic health records (EHR) of all patients aged 18 years and older for whom a RC was requested. We excluded patients with incomplete records or if the consultation concerned a previously diagnosed IRD, resulting in a final cohort of 340 patients.

### Variables

The EHR of ED patients were manually reviewed. We collected a broad range of predefined parameters (Supplementary Table 1). Patients with ambiguous data points were reviewed by a senior rheumatologist (S.M.). For the presenting complaints we used a bottom-up approach. We first identified all symptoms that led to ED presentation. These were then combined and categorized based on frequency and relevancy to IRDs (Supplementary Table 2). Classification of urgency of therapy was done by ED staff according to the MTS in a standardized manner: T1: Immediate (0 min), T2: very urgent (10 min), T3: urgent (30 min), T4: less urgent (90 min), T5: non-urgent (120 min) [5]. Pain severity was assessed in a standardized manner at arrival and graded on a scale from 0 (no pain) to 10 (most severe imaginable pain). All other variables (e.g. symptom duration) were extracted from the EHR. Final diagnoses were determined by the clinical teams managing the patients. There was no follow up of patients after this point and formal classification criteria were not followed.

### Ethics

A formal ethics application was submitted to the local ethics board. In view of the fact that the patient data that were the subject of the study can no longer be attributed to a human being (anonymized) this study didn’t constitute a "research project involving human beings" as defined in Section 9 (2) of the Hamburg Chamber Act for the Medical Professions and also did not fall within the scope of the research projects requiring consultation pursuant to Section 15 (1) of the Professional Code of Conduct for Hamburg Physicians. The study thus received a waiver by the ethics board.

### Statistical analysis

Statistical analyses were performed using Graph Prism (v10.2.3, GraphPad Prism Software, Boston, MA, USA). A multiple logistic regression analysis was performed. The dependent variable was “discharge diagnosis of any IRD” (yes/no). We included all demographic and process variables and included the ten most common presenting symptoms as well as typical rheumatological symptoms (Supplementary Table 3). The clinical impression/diagnosis of the consulting rheumatologist was added to the other factors in a second analysis (Supplementary Table 4).

### Neural network

The R package “neuralnet” (version 1.44.2) was employed for creating a neural network using R (version 4.5.0). We randomly divided patient data into two sets, resulting in a set of training data (70%, 234 patients) and a set of test data (30%, 105 patients). Training data was used to construct our neural network using resilient backpropagation with backtracking and the neural network was subsequently tested on our set of test data. No external validation was performed. The parameters for the neural network were the same as were used for the logistic regression.

## Results

### Patients with a susIRD present with a wide range of symptoms

**Fig. 1:**
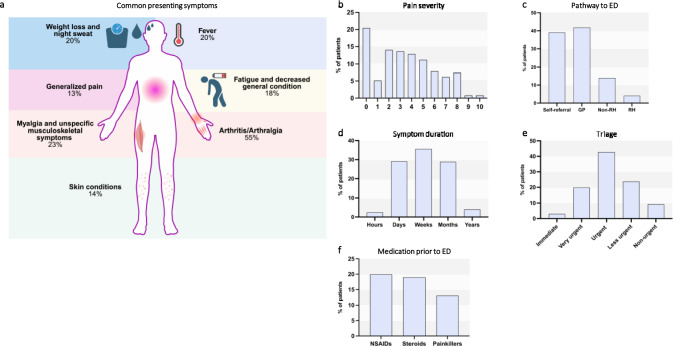
Patient spectrum in the Emergency Department. (**a**) Diagram of the 7 most common symptoms / symptom-complexes that lead to presentation to the ED (emergency department). The percentage of patients presenting with the symptom is indicated. (**b**) Pain severity at presentation to the ED. Pain severity is measured by numeric rating scale (NRS). (**c**) Pathways to the ED. Patients presented on their own initiative (Self-referral) or after referral from either a general practitioner/family doctor (GP), a rheumatologist (RH) or another medical specialty (Non-RH). (**d**) Duration of symptoms is categorized according to whether symptoms persisted for hours (0-24 h), days (1-7d), weeks (1-4w), months (1-12 m) or years (> 12 m) at time of presentation. (**e**) Urgency of medical attention was triaged according to Manchester-Triage-System (MTS) and is categorized as immediate, very urgent, urgent, less urgent or non-urgent. (**f**) Depiction of select medication patients were already taking when presenting to ED. Non-Steroidal Anti-Inflammatory Drugs (NSAIDs). Created in BioRender. P. Klimivecs (2026) https://BioRender.com/wxtpq38

We identified a total of 340 patients where the ED-team had requested a RC for evaluation of a patient because they suspected an IRD. These patients were defined as susIRD in the context of our study. For quality control 40 patient charts (12% of the total cohort) were blindly assessed by two investigators. Inter-rater reliability was 95%. We analyzed the symptoms that led to ED presentation and found a total of 50 different symptom-complexes (Supplementary Table 2). Arthritis/arthralgias (55%) were the most common presenting complaint, followed by myalgia and unspecific musculoskeletal (MSK) symptoms (23%), weight loss and night sweats (20%), fever (20%), fatigue and decreased general condition (DGC) (18%), skin conditions (14%) and generalized pain (13%) (Fig. 1a). However, 13% of patients did not have any of these 7 symptoms. Pain is generally a very common reason for ED presentation. Overall, 87% of patients complained of pain. The mean severity of pain of all patients was 3.4 (standard deviation 2.6) on a 10-point numeric rating scale (Fig. 1b).

### Most susIRD patients don’t present as true emergencies

Next, we analyzed various general aspects of the mode of presentation. Overall, 42% of patients were seen by their general practitioner (GP) and referred to the ED (Fig. 1c). A further 14% had been seen by another medical specialist in the community. Only 5% had been seen by a rheumatologist. In contrast, 39% of patients presented directly to the ED without consulting any doctor. Consistent with this, the majority presented as walk-in patients (82%) with the remaining 18% presenting via ambulance. In line, most patients (83%) presented within normal working hours (8am to 6 pm; Monday to Friday). Only 2.5% of patients had symptoms lasting 0–24 h at the time of ED presentation (Fig. 1d). Symptom duration for days (1–7 d), weeks (1–4 w), months (1–12 m) and years (> 12 m) was 29%, 36%, 29% and 4% respectively. Despite the often-prolonged symptom duration, patients were triaged as being relatively severely ill. Overall, 66% of patients were triaged as at least urgent and only 9% were deemed to be non-urgent (Fig. 1e).

We found elevated inflammatory markers with a mean (SD) C-reactive protein (CRP) of 78 mg/l (76). Prior to presentation to the ED, painkillers and non-steroidal anti-inflammatory drugs (NSAIDs) had only been taken by 13% and 20% respectively. Surprisingly, 19% of patients had been given corticosteroids (CS) prior to ED presentation (Fig. 1f).

### RC requests are in line with presenting symptoms

**Fig. 2:**
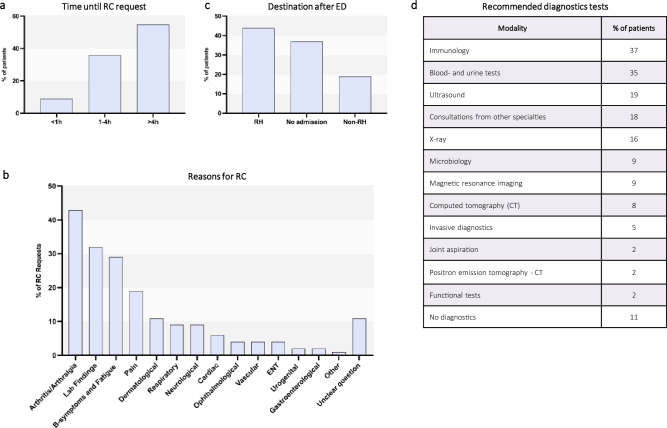
Rheumatological consultation. (**a**) Depiction of the time it took after initial presentation to the ED (emergency department) until rheumatological consultation (RC) was requested by the attending physician. (**b**) Symptoms and findings that led to RC. (**c**) Overview of further diagnostic testing recommended by RC. (**d**) Patient Pathways after the ED. Patients were admitted either under rheumatology (RH) or under another specialty (Non-RH) or discharged from ED (No admission)

The time span between presentation to the ED and the RC-request was < 1 h, 1-4 h and > 4 h in 9%, 36% and 55%, respectively. This suggests that the ED doctors were not initially considering rheumatological diseases (Fig. 2a). Despite this, 48% of RC requests were labelled as urgent.

Next, we analyzed the reasons for which RCs were requested by the ED team. As expected, arthritis/arthralgias was the most common reason for RC (43%) with fatigue and DGC (29%) and pain (19%) also being common. Abnormal lab findings (e.g. elevated inflammatory markers such as CRP) were also very prevalently given as reasons for RC (32%) (Fig. 2b). In addition, RC requests were prompted by abnormalities affecting multiple organ systems, including dermatological (11%), respiratory (9%), neurological (9%), and cardiac manifestations (6%) (Fig. 2b).

### RCs have a great impact on patient management

The recommendations of the consulting rheumatologists included a broad range of diagnostic modalities (Fig. 2c). Immunological and specific rheumatological tests (37%) as well as other lab tests (35%) were the most common recommendations. Consultation by other specialties (18%) was also commonly advised. As expected, a wide range of imaging was recommended, including ultrasound (19%), plain X-rays (16%), MRI (9%), CT (8%) and even PET-CT (2%). Advice for hospital admission or rheumatological outpatient appointment was given in 33% and 21% of cases respectively.

Following from this, we analyzed the actual treatment pathways of the susIRD patients. The majority were admitted to the hospital, either under the care of rheumatology (44%) or another medical specialty (19%) (Fig. 2d). The remaining patients (37%) were discharged from the ED. The RC majorly shaped patient pathways. When the RC recommended outpatient management, 92% (49/53) of susIRD patients were discharged from the ED, indicating that the RC majorly affected treatment pathways.

### An IRD was diagnosed in 62% of patients

**Fig. 3:**
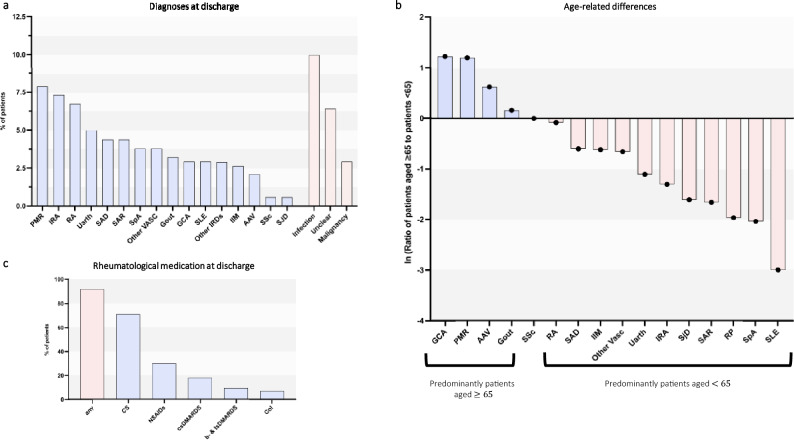
Rheumatological diagnoses and treatment. (**a**) Overview of diagnoses at discharge from hospital. (**b**) Age-dependent presentation of selected diagnoses. Ratios of Frequencies of patients older and younger than 65 years for the depicted diagnoses with Woolf-Haldane correction. (**c**) Overview of rheumatological medication at discharge. Polymyalgia rheumatica (PMR), reactive arthritis/parainfectious arthritis (IRA), rheumatoid arthritis (RA), unspecified active arthritis (Uarth), systemic autoinflammatory disease (SAD), sarcoidosis (SAR), spondyloarthritis (SpA), other vasculitis (Other VASC), giant cell arteritis (GCA), systemic lupus erythematosus (SLE), inflammatory rheumatic disease (IRD), idiopathic inflammatory myopathies (IIM), ANCA-associated vasculitis (AAV), systemic sclerosis (SSc), Sjögren’s disease (SJD), corticosteroids (CS), non-steroidal anti-inflammatory drugs (NSAIDs), conventional synthetic disease-modifying antirheumatic drugs (csDMARDs), biological- and targeted synthetic disease-modifying antirheumatic drugs (b- & tsDMARDs), colchicine (Col)

At discharge, a clinical diagnosis of a new IRD was made in 62% of susIRD patients. There was a broad spectrum of newly diagnosed rheumatological diseases (Fig. 3a), with polymyalgia rheumatica (PMR) [7.9%], reactive arthritis/para infectious arthritis (IRA) [7.3%] and rheumatoid arthritis (RA) [6.3%] the most prevalent. The most common non-IRD diagnosis was infection (10.0%). The mean age of patients with a newly diagnosed IRD was 54 years. We compared the frequencies of specific rheumatological diagnoses in patients older or younger than 65 years of age (Fig. 3b). As expected, PMR, GCA and anti-neutrophil cytoplasmic antibody associated vasculitis (AAV) were more common in patients > 65 years. Anti-Inflammatory and immunosuppressive therapy was started in 92% of patients with a diagnosed IRD (Fig. 3c). The majority (71%) were treated with corticosteroids. NSAIDs (30%), csDMARDS (18%), colchicine (7%) and b- or tsDMARDS (9.5%; mostly anti-IL6 therapy) were also prescribed (Fig. 3c).

### Clinical parameters predict the diagnoses of IRD with low accuracy

**Fig. 4:**
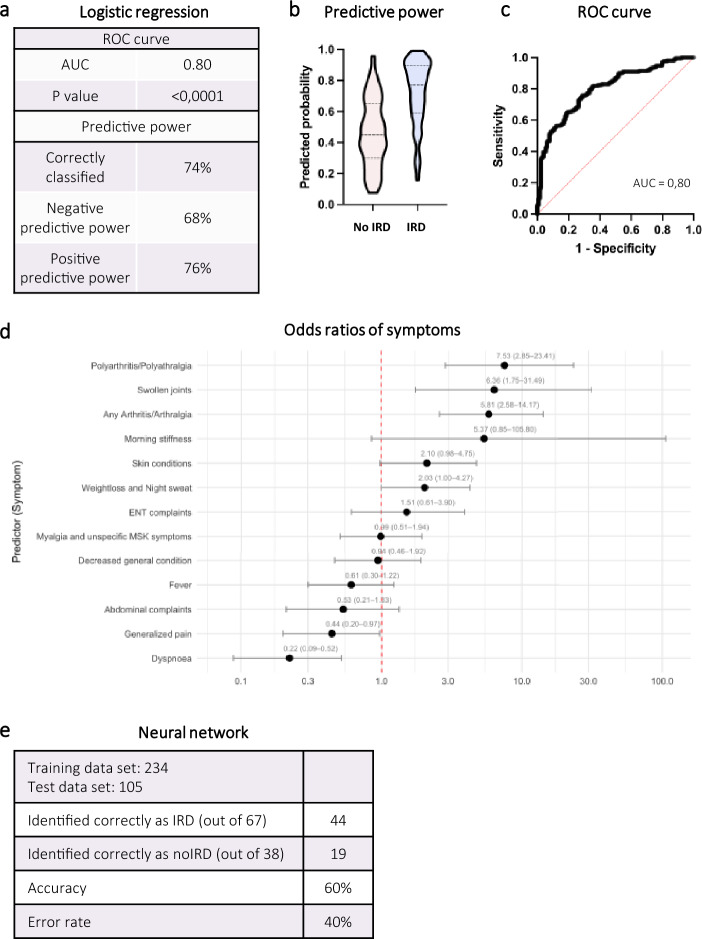
Prediction tools. (**a**) Key attributes of the logistic regression model for predicting an inflammatory rheumatic disease (IRD) diagnosis. Receiver-operation characteristic curve (ROC), Area under the curve (AUC). (**b**) Predicted probability of an eventual IRD diagnosis or not (no IRD) and (**c**) depiction of ROC curve for the logistic regression model. (**d**) Association of individual symptoms with an eventual IRD diagnosis by odds ratios (OR) derived from the logistic regression model. (**e**) Outcome and key metrics of our neural network without (w/o) rheumatological consultation (RC) as an input parameter

Next, we sought to determine if we can predict which susIRD patients would be diagnosed with an IRD. Therefore, we developed a logistic regression model incorporating 24 presentation-related parameters (Supplementary Table 3). This model showed moderate accuracy. It correctly classified 74% of patients (negative predictive power 68%; positive predictive power 76%) (Fig. 4a, b). The area under the receiver operating characteristic curve (ROC curve) was 0.80 (Fig. 4a, c). The significant positive predictors were polyarthritis/polyarthralgia (OR 7.5), any arthritis/arthralgias (OR 5.8) and swollen joints (OR 6.4). Unspecific pain (OR 0.44) and dyspnea (OR 0.22) were significant negative predictors (Fig. 4d).

As an additional method to predict IRD diagnosis we employed a neural network using the aforementioned parameters (Supplementary Fig. 1). The neural network correctly identified 60% of cases (Fig. 4e).

### Rheumatologists accurately diagnose IRDs in the ED and greatly improve the accuracy of prediction models

**Fig. 5:**
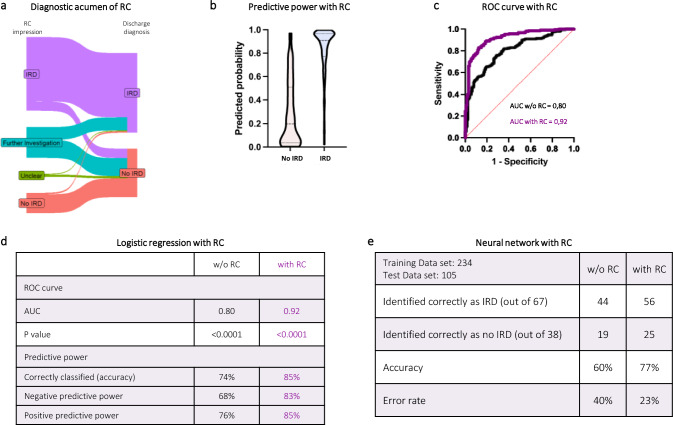
Rheumatological expertise in the ED (Emergency department). (**a**) Visualization of initially suspected diagnosis by rheumatological consultation (RC) and eventual diagnosis at discharge. IRD: Inflammatory rheumatic disease. (**b**) Predicted probability of an eventual IRD diagnosis or no IRD with RC of the logistic regression model with RC. (**c**) Comparison of area under the ROC curve (AUC) for the logistic regression model with and without (w/o) RC. (**d**) Comparison of key attributes of the logistic regression model with and w/o RC. (**e**) Comparison of key attributes of the neural network with and w/o RC

Next, we analyzed the impact of rheumatological expertise in the ED. Rheumatologists demonstrated high accuracy in diagnosing IRD in the ED setting (Fig. 5a). When the consulting rheumatologist suspected an IRD, this was confirmed in 87% of patients during the admission. The negative predictive value (NPV) was also high; when the consulting rheumatologist deemed an IRD as unlikely, only 7% of patients were eventually diagnosed with an IRD during the admission.

Finally, we analyzed the combination of our prediction models and rheumatological expertise. RCs greatly increased the predictive power of the logistic regression model (Fig. 5b-d, Supplementary Table ST4). The accuracy increased from 74 to 85%. The odds ratio for “rheumatologist suspects IRD” was 126, far exceeding that of all other predictors. The accuracy of the neural network was also greatly increased, going from 60 to 77% (Fig. 5e).

## Discussion

We aimed to characterize the spectrum of rheumatological patients in the ED without a prior IRD diagnosis. Whilst inflammatory musculoskeletal conditions (especially arthritis) are a major aspect of rheumatology, patients with IRDs can present with a multitude of different symptoms and involvement of different organ systems. Fittingly, the most common complaint in our cohort was arthritis/arthralgias, but patients presented with a diverse range of symptoms (Fig. 1a, Supplementary Table 2). In line with this, the most common reasons for RCs were arthritis/arthralgia, lab findings and constitutional symptoms, although many other organ systems were represented (Fig. 2b). Previous data on patients with known IRDs have also shown high proportions (37%) of arthritis [18].

There is an important distinction between true medical emergencies (e.g. diffuse pulmonary hemorrhage) and not immediately life-threatening medical urgencies (e.g. acute gout flare). According to the WHO definition in the former case failure to provide immediate care may lead to the death of the patient in a matter of minutes [19]. A Canadian study looked at ED utilization of patients with known rheumatoid arthritis and found half the visits to be classified as “less urgent” or “non-urgent” [20].

We thus wanted to assess disease duration and severity of our patients. Our data indicates that most patients presented as urgencies rather than true emergencies; walk-ins accounted for 82% of susIRD patients and 83% presented during normal working hours (Fig. 1d-e). Furthermore, symptoms had been present for at least 7 days in about two thirds of patients with only a small minority presenting within hours of symptom onset (Fig. 1f). This implies that patients could have been assessed and treated in the community as outpatients. This is in line with data from San et al. where less urgent (52.5%) and non-urgent (19.9%) patients accounted for the majority of ED patients [2]. However, our patients were classified as severely ill; 65% were triaged as urgent or higher by MTS (Fig. 1e). Similarly, ED doctors categorized 48% of RC requests as urgent. A Canadian study investigating ED utilization of patients with known IRD reported that a significant proportion of patients (29%) proceeded directly to the ED, without prior consultation of their rheumatologist or primary care doctor. Furthermore, 35% of patients had attempted to access care from one or more providers in the community prior to presenting to the ED [18]. Another study showed that, on average, patients wait more than three months for their first outpatient rheumatology appointment. This is despite a recommended quality target of consultation within six weeks of symptom onset [21–23]. In a separate analysis, 25% of patients with RA had symptoms for longer than a year before receiving a diagnosis. In axial spondylarthritis (axSpA) the rate was even higher at 58% [24]. A plausible explanation for the discrepancy in our data could be worsening during prolonged waiting periods for community-based care with the eventual need for ED and hospital treatment. This is supported by the fact that almost two-thirds of patients with susIRD were admitted to the hospital (Fig. 2d). This indicates suboptimal patient care as well as ineffective use of the ED which is an expensive resource in the healthcare system. Worldwide healthcare systems are under increasing stress to optimize resource utilization [25]. Whilst there are many other ways to assess healthcare resource utilization (HCRU), the ED is positioned at a critical point at the interface between community and in-patient care. We believe that our study is unique in its detailed analysis of rheumatological ED patients and identifies potential targets for future research for optimization of HCRU by rheumatological patients. In particular, clearer referral pathways and earlier access to rheumatological care in the outpatient setting may reduce reliance on the ED for initial IRD assessment.

Whilst use of NSAIDs and other pain medication prior to ED presentation is to be expected we also found a surprisingly high proportion (19%) of patients who had been given CS prior to ED presentation (Fig. 1f). This finding is consistent with data from Fuchs et al. [26] found that 32.2% of IRD patients received medical treatment that eased their symptoms prior to rheumatology appointments. The high rate of CS use prior to definitive rheumatological diagnosis is alarming and likely reflects delayed access to specialist care. Empirical CS therapy may temporarily alleviate symptoms but risks masking disease activity, complicating diagnostic assessment, and postponing the initiation of targeted, guideline-based therapy.

In 62% of susIRD patients a diagnosis of an IRD could be made. In addition to relatively common conditions (PMR, RA) we diagnosed a substantial number of rare diseases (Fig. 2a). Employing a multiple logistic regression model with 24 clinical parameters, we aimed to identify parameters that could predict the diagnosis of an IRD in our study population. Our model had moderate to low predictive power with an accuracy of only 74% and an AUC of 0.80 (Fig. 4a-c). Not surprisingly, arthritis/arthralgias were the strongest predictor of an IRD diagnosis (Fig. 4d). The multiple non-linear layers of a neural network allow it to automatically learn and represent intricate, non-linear relationships and interactions between inputs giving it a potential advantage over logistic regression. However, our neural network only showed relatively low accuracy (Fig. 4e). It should be noted that the logistic regression model was evaluated on the total cohort, whereas the neural network performance was assessed on a hold-out test set, which may account for the observed difference in accuracy. Overall, based on our input data, neither the multiple logistic regression model nor the neural network were accurate enough to be used in a clinical setting. However, in the right context, decision support systems can be used successfully for rheumatic conditions [27]; In a recent paper on diagnostic accuracy of large language models in the ED a high degree of accuracy was found. However, none of the patients were classified as having symptoms suggesting a rheumatic disease and only 3% had pain as the chief complaint [28].

There is data that RCs for hospitalized patients significantly enhance the diagnostic accuracy and adherence to established guidelines for short- and long-term treatment for some conditions [13]. In our study rheumatologists gave detailed advice on further investigations (Fig. 2c). RCs substantially influenced patient pathways. When outpatient management was recommended, 92% of patients were indeed discharged from the ED.

Importantly, the consulting rheumatologists were highly accurate in predicting the eventual diagnoses of susIRD patients (Fig. 5a). Whilst we did not have access to hard outcome data (e.g. mortality), we believe that the increased diagnostic accuracy of the RC is likely leading to better management.

The incorporation of the impression of the RC also markedly improved the accuracy of our prediction models with the logistic regression increasing from 74 to 85% and the neural network from 60 to 77%.

There are several limitations to our study. Firstly, idiosyncratic aspects of the German health care system as well as institution-specific factors, make generalization to other settings difficult. As an example, a same-day emergency care service in London, UK, could deliver effective care to rheumatological urgencies [29].

For example, dedicated urgent walk-in rheumatology appointments could have substantially shifted patient pathways. Secondly, our cohort includes only patients for whom ED physicians requested a RC. A major limitation of our study is the reliance on RC to identify patients. We believe that, given the structure of our ED, this should identify most patients with potential IRDs but it does introduce significant bias as patients that were never presented to the rheumatology team might eventually have been diagnosed with an IRD.

In addition, the retrospective design and reliance on electronic health record documentation limit data completeness. Lastly, we did no follow-up of patients after discharge and can thus not be sure that diagnoses were not revised at a later stage. Prospective, multicenter studies are therefore needed to validate and extend our findings.

## Conclusion

We characterized the spectrum of patients with suspected new IRD in the ED. Most patients were medical urgencies rather than true emergencies, demonstrating the need for further research into optimization of HCRU. Our prediction models demonstrated only moderate accuracy in identifying patients who were ultimately diagnosed with an IRD. Rheumatologists were substantially more accurate, indicating the need for rheumatological expertise in the ED. Overall, we believe that our data adds significantly to the understanding of rheumatological patients in the ED and the role of the rheumatologist in emergency medicine.

## Supplementary Information

Below is the link to the electronic supplementary material.

Supplementary file1 (PPTX 773 KB)
