# Supplementary material for: Clinical spectrum and diagnostic outcomes of patients with suspected inflammatory rheumatic disease in the emergency department: a retrospective study
Source: Rheumatol Int. 2026 Jun 5;46(6):136. doi: 10.1007/s00296-026-06113-4 (PMC13241444; doi:10.1007/s00296-026-06113-4)
Supplement: Supplementary file 1 — Supplementary file1 (PPTX 773 KB) [file 296_2026_6113_MOESM1_ESM.pptx]

## Slide 1
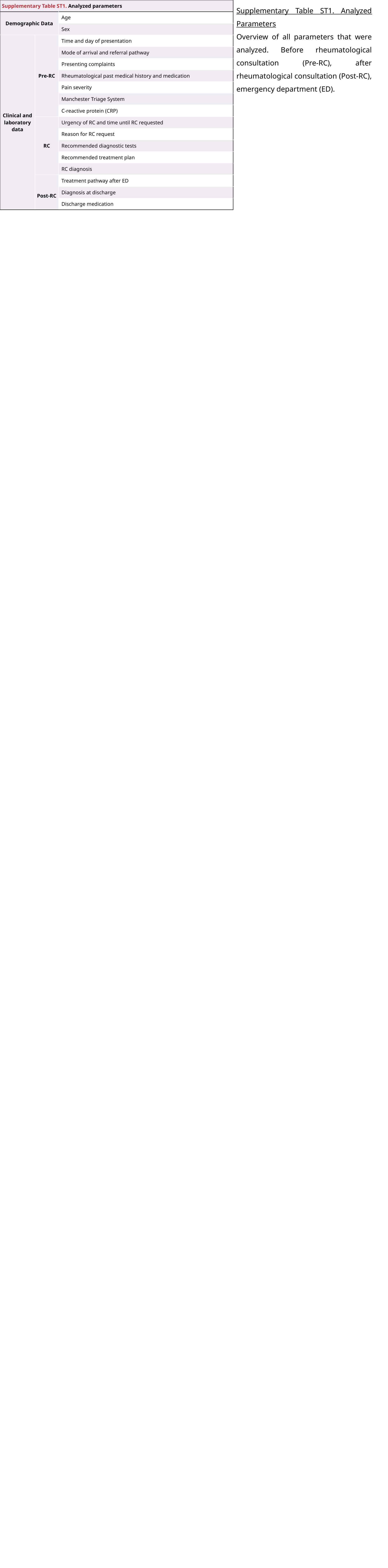

| Supplementary Table ST1. Analyzed parameters | | |
| --- | --- | --- |
| Demographic Data | | Age |
| | | Sex |
| Clinical and laboratory data | Pre-RC | Time and day of presentation |
| | | Mode of arrival and referral pathway |
| | | Presenting complaints |
| | | Rheumatological past medical history and medication |
| | | Pain severity |
| | | Manchester Triage System |
| | | C-reactive protein (CRP) |
| | RC | Urgency of RC and time until RC requested |
| | | Reason for RC request |
| | | Recommended diagnostic tests |
| | | Recommended treatment plan |
| | | RC diagnosis |
| | Post-RC | Treatment pathway after ED |
| | | Diagnosis at discharge |
| | | Discharge medication |
Supplementary Table ST1. Analyzed Parameters
Overview of all parameters that were analyzed. Before rheumatological consultation (Pre-RC), after rheumatological consultation (Post-RC), emergency department (ED).

## Slide 2
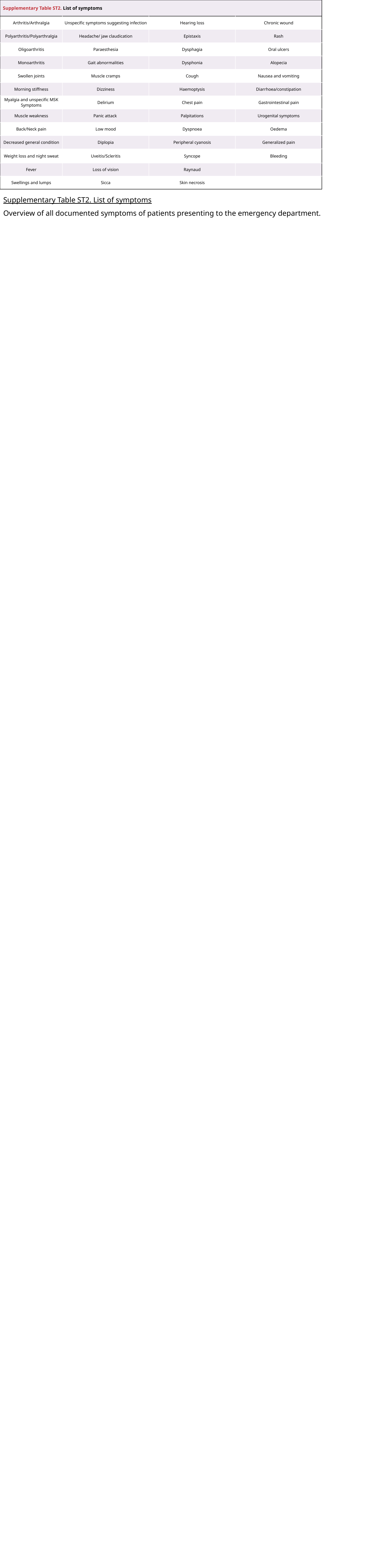

| Supplementary Table ST2. List of symptoms | | | |
| --- | --- | --- | --- |
| Arthritis/Arthralgia | Unspecific symptoms suggesting infection | Hearing loss | Chronic wound |
| Polyarthritis/Polyarthralgia | Headache/ jaw claudication | Epistaxis | Rash |
| Oligoarthritis | Paraesthesia | Dysphagia | Oral ulcers |
| Monoarthritis | Gait abnormalities | Dysphonia | Alopecia |
| Swollen joints | Muscle cramps | Cough | Nausea and vomiting |
| Morning stiffness | Dizziness | Haemoptysis | Diarrhoea/constipation |
| Myalgia and unspecific MSK Symptoms | Delirium | Chest pain | Gastrointestinal pain |
| Muscle weakness | Panic attack | Palpitations | Urogenital symptoms |
| Back/Neck pain | Low mood | Dyspnoea | Oedema |
| Decreased general condition | Diplopia | Peripheral cyanosis | Generalized pain |
| Weight loss and night sweat | Uveitis/Scleritis | Syncope | Bleeding |
| Fever | Loss of vision | Raynaud | |
| Swellings and lumps | Sicca | Skin necrosis | |
Supplementary Table ST2. List of symptoms
Overview of all documented symptoms of patients presenting to the emergency department.

## Slide 3
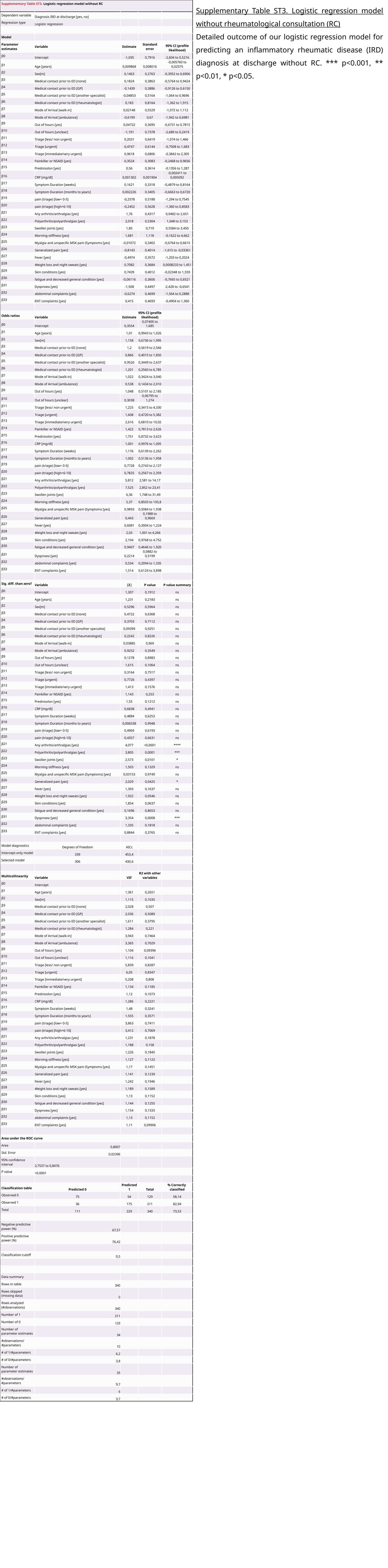

| Supplementary Table ST3. Logistic regression model without RC | | | | |
| --- | --- | --- | --- | --- |
| Dependent variable | Diagnosis IRD at discharge [yes, no] | | | |
| Regression type | Logistic regression | | | |
| | | | | |
| Model | | | | |
| Parameter estimates | Variable | Estimate | Standard error | 95% CI (profile likelihood) |
| β0 | Intercept | -1,035 | 0,7916 | -2,604 to 0,5216 |
| β1 | Age [years] | 0,009868 | 0,008016 | -0,005760 to 0,02575 |
| β2 | Sex[m] | 0,1463 | 0,2763 | -0,3952 to 0,6906 |
| β3 | Medical contact prior to ED [none] | 0,1824 | 0,3863 | -0,5764 to 0,9424 |
| β4 | Medical contact prior to ED [GP] | -0,1439 | 0,3886 | -0,9126 to 0,6150 |
| β5 | Medical contact prior to ED [another specialist] | -0,04853 | 0,5164 | -1,064 to 0,9696 |
| β6 | Medical contact prior to ED [rheumatologist] | 0,183 | 0,8164 | -1,362 to 1,915 |
| β7 | Mode of Arrival [walk-in] | 0,02148 | 0,5529 | -1,072 to 1,112 |
| β8 | Mode of Arrival [ambulance] | -0,6199 | 0,67 | -1,942 to 0,6981 |
| β9 | Out of hours [yes] | 0,04722 | 0,3695 | -0,6731 to 0,7815 |
| β10 | Out of hours [unclear] | -1,191 | 0,7378 | -2,689 to 0,2419 |
| β11 | Triage [less/ non urgent] | 0,2031 | 0,6419 | -1,074 to 1,466 |
| β12 | Triage [urgent] | 0,4747 | 0,6144 | -0,7508 to 1,683 |
| β13 | Triage [immediate/very urgent] | 0,9618 | 0,6806 | -0,3842 to 2,305 |
| β14 | Painkiller or NSAID [yes] | 0,3524 | 0,3083 | -0,2468 to 0,9656 |
| β15 | Prednisolon [yes] | 0,56 | 0,3614 | -0,1356 to 1,287 |
| β16 | CRP [mg/dl] | 0,001302 | 0,001904 | -0,002411 to 0,005092 |
| β17 | Symptom Duration [weeks] | 0,1621 | 0,3318 | -0,4879 to 0,8164 |
| β18 | Symptom Duration [months to years] | 0,002226 | 0,3405 | -0,6663 to 0,6720 |
| β19 | pain (triage) [low= 0-5] | -0,2578 | 0,5188 | -1,294 to 0,7545 |
| β20 | pain (triage) [high=6-10] | -0,2452 | 0,5628 | -1,360 to 0,8583 |
| β21 | Any arthritis/arthralgias [yes] | 1,76 | 0,4317 | 0,9482 to 2,651 |
| β22 | Polyarthritis/polyarthralgias [yes] | 2,018 | 0,5304 | 1,048 to 3,153 |
| β23 | Swollen joints [yes] | 1,85 | 0,719 | 0,5584 to 3,450 |
| β24 | Morning stiffness [yes] | 1,681 | 1,118 | -0,1622 to 4,662 |
| β25 | Myalgia and unspecific MSK pain (Symptoms [yes] | -0,01072 | 0,3402 | -0,6764 to 0,6615 |
| β26 | Generalized pain [yes] | -0,8143 | 0,4014 | -1,615 to -0,03361 |
| β27 | Fever [yes] | -0,4974 | 0,3572 | -1,203 to 0,2024 |
| β28 | Weight loss and night sweats [yes] | 0,7082 | 0,3684 | 0,0008233 to 1,451 |
| β29 | Skin conditions [yes] | 0,7439 | 0,4012 | -0,02348 to 1,559 |
| β30 | fatigue and decreased general condition [yes] | -0,06116 | 0,3606 | -0,7665 to 0,6521 |
| β31 | Dyspnoea [yes] | -1,508 | 0,4497 | -2,428 to -0,6541 |
| β32 | abdominal complaints [yes] | -0,6274 | 0,4699 | -1,564 to 0,2888 |
| β33 | ENT complaints [yes] | 0,415 | 0,4693 | -0,4904 to 1,360 |
| | | | | |
| Odds ratios | Variable | Estimate | 95% CI (profile likelihood) | |
| β0 | Intercept | 0,3554 | 0,07400 to 1,685 | |
| β1 | Age [years] | 1,01 | 0,9943 to 1,026 | |
| β2 | Sex[m] | 1,158 | 0,6736 to 1,995 | |
| β3 | Medical contact prior to ED [none] | 1,2 | 0,5619 to 2,566 | |
| β4 | Medical contact prior to ED [GP] | 0,866 | 0,4015 to 1,850 | |
| β5 | Medical contact prior to ED [another specialist] | 0,9526 | 0,3449 to 2,637 | |
| β6 | Medical contact prior to ED [rheumatologist] | 1,201 | 0,2560 to 6,785 | |
| β7 | Mode of Arrival [walk-in] | 1,022 | 0,3424 to 3,040 | |
| β8 | Mode of Arrival [ambulance] | 0,538 | 0,1434 to 2,010 | |
| β9 | Out of hours [yes] | 1,048 | 0,5101 to 2,185 | |
| β10 | Out of hours [unclear] | 0,3038 | 0,06795 to 1,274 | |
| β11 | Triage [less/ non urgent] | 1,225 | 0,3415 to 4,330 | |
| β12 | Triage [urgent] | 1,608 | 0,4720 to 5,382 | |
| β13 | Triage [immediate/very urgent] | 2,616 | 0,6810 to 10,02 | |
| β14 | Painkiller or NSAID [yes] | 1,422 | 0,7813 to 2,626 | |
| β15 | Prednisolon [yes] | 1,751 | 0,8732 to 3,623 | |
| β16 | CRP [mg/dl] | 1,001 | 0,9976 to 1,005 | |
| β17 | Symptom Duration [weeks] | 1,176 | 0,6139 to 2,262 | |
| β18 | Symptom Duration [months to years] | 1,002 | 0,5136 to 1,958 | |
| β19 | pain (triage) [low= 0-5] | 0,7728 | 0,2743 to 2,127 | |
| β20 | pain (triage) [high=6-10] | 0,7825 | 0,2567 to 2,359 | |
| β21 | Any arthritis/arthralgias [yes] | 5,812 | 2,581 to 14,17 | |
| β22 | Polyarthritis/polyarthralgias [yes] | 7,525 | 2,852 to 23,41 | |
| β23 | Swollen joints [yes] | 6,36 | 1,748 to 31,49 | |
| β24 | Morning stiffness [yes] | 5,37 | 0,8503 to 105,8 | |
| β25 | Myalgia and unspecific MSK pain (Symptoms [yes] | 0,9893 | 0,5084 to 1,938 | |
| β26 | Generalized pain [yes] | 0,443 | 0,1988 to 0,9669 | |
| β27 | Fever [yes] | 0,6081 | 0,3004 to 1,224 | |
| β28 | Weight loss and night sweats [yes] | 2,03 | 1,001 to 4,266 | |
| β29 | Skin conditions [yes] | 2,104 | 0,9768 to 4,752 | |
| β30 | fatigue and decreased general condition [yes] | 0,9407 | 0,4646 to 1,920 | |
| β31 | Dyspnoea [yes] | 0,2214 | 0,0882 to 0,5199 | |
| β32 | abdominal complaints [yes] | 0,534 | 0,2094 to 1,335 | |
| β33 | ENT complaints [yes] | 1,514 | 0,6124 to 3,898 | |
| | | | | |
| Sig. diff. than zero? | Variable | |Z| | P value | P value summary |
| β0 | Intercept | 1,307 | 0,1912 | ns |
| β1 | Age [years] | 1,231 | 0,2183 | ns |
| β2 | Sex[m] | 0,5296 | 0,5964 | ns |
| β3 | Medical contact prior to ED [none] | 0,4722 | 0,6368 | ns |
| β4 | Medical contact prior to ED [GP] | 0,3703 | 0,7112 | ns |
| β5 | Medical contact prior to ED [another specialist] | 0,09399 | 0,9251 | ns |
| β6 | Medical contact prior to ED [rheumatologist] | 0,2242 | 0,8226 | ns |
| β7 | Mode of Arrival [walk-in] | 0,03885 | 0,969 | ns |
| β8 | Mode of Arrival [ambulance] | 0,9252 | 0,3549 | ns |
| β9 | Out of hours [yes] | 0,1278 | 0,8983 | ns |
| β10 | Out of hours [unclear] | 1,615 | 0,1064 | ns |
| β11 | Triage [less/ non urgent] | 0,3164 | 0,7517 | ns |
| β12 | Triage [urgent] | 0,7726 | 0,4397 | ns |
| β13 | Triage [immediate/very urgent] | 1,413 | 0,1576 | ns |
| β14 | Painkiller or NSAID [yes] | 1,143 | 0,253 | ns |
| β15 | Prednisolon [yes] | 1,55 | 0,1212 | ns |
| β16 | CRP [mg/dl] | 0,6838 | 0,4941 | ns |
| β17 | Symptom Duration [weeks] | 0,4884 | 0,6253 | ns |
| β18 | Symptom Duration [months to years] | 0,006538 | 0,9948 | ns |
| β19 | pain (triage) [low= 0-5] | 0,4969 | 0,6193 | ns |
| β20 | pain (triage) [high=6-10] | 0,4357 | 0,6631 | ns |
| β21 | Any arthritis/arthralgias [yes] | 4,077 | <0,0001 | \*\*\*\* |
| β22 | Polyarthritis/polyarthralgias [yes] | 3,805 | 0,0001 | \*\*\* |
| β23 | Swollen joints [yes] | 2,573 | 0,0101 | \* |
| β24 | Morning stiffness [yes] | 1,503 | 0,1329 | ns |
| β25 | Myalgia and unspecific MSK pain (Symptoms) [yes] | 0,03153 | 0,9749 | ns |
| β26 | Generalized pain [yes] | 2,029 | 0,0425 | \* |
| β27 | Fever [yes] | 1,393 | 0,1637 | ns |
| β28 | Weight loss and night sweats [yes] | 1,922 | 0,0546 | ns |
| β29 | Skin conditions [yes] | 1,854 | 0,0637 | ns |
| β30 | fatigue and decreased general condition [yes] | 0,1696 | 0,8653 | ns |
| β31 | Dyspnoea [yes] | 3,354 | 0,0008 | \*\*\* |
| β32 | abdominal complaints [yes] | 1,335 | 0,1818 | ns |
| β33 | ENT complaints [yes] | 0,8844 | 0,3765 | ns |
| | | | | |
| Model diagnostics | Degrees of Freedom | AICc | | |
| Intercept-only model | 339 | 453,4 | | |
| Selected model | 306 | 430,6 | | |
| | | | | |
| Multicollinearity | Variable | VIF | R2 with other variables | |
| β0 | Intercept | | | |
| β1 | Age [years] | 1,361 | 0,2651 | |
| β2 | Sex[m] | 1,115 | 0,1035 | |
| β3 | Medical contact prior to ED [none] | 2,028 | 0,507 | |
| β4 | Medical contact prior to ED [GP] | 2,036 | 0,5089 | |
| β5 | Medical contact prior to ED [another specialist] | 1,611 | 0,3795 | |
| β6 | Medical contact prior to ED [rheumatologist] | 1,284 | 0,221 | |
| β7 | Mode of Arrival [walk-in] | 3,943 | 0,7464 | |
| β8 | Mode of Arrival [ambulance] | 3,365 | 0,7029 | |
| β9 | Out of hours [yes] | 1,104 | 0,09396 | |
| β10 | Out of hours [unclear] | 1,116 | 0,1041 | |
| β11 | Triage [less/ non urgent] | 5,839 | 0,8287 | |
| β12 | Triage [urgent] | 6,05 | 0,8347 | |
| β13 | Triage [immediate/very urgent] | 5,208 | 0,808 | |
| β14 | Painkiller or NSAID [yes] | 1,134 | 0,1185 | |
| β15 | Prednisolon [yes] | 1,12 | 0,1073 | |
| β16 | CRP [mg/dl] | 1,286 | 0,2221 | |
| β17 | Symptom Duration [weeks] | 1,48 | 0,3241 | |
| β18 | Symptom Duration [months to years] | 1,555 | 0,3571 | |
| β19 | pain (triage) [low= 0-5] | 3,863 | 0,7411 | |
| β20 | pain (triage) [high=6-10] | 3,412 | 0,7069 | |
| β21 | Any arthritis/arthralgias [yes] | 1,231 | 0,1878 | |
| β22 | Polyarthritis/polyarthralgias [yes] | 1,188 | 0,158 | |
| β23 | Swollen joints [yes] | 1,226 | 0,1845 | |
| β24 | Morning stiffness [yes] | 1,127 | 0,1123 | |
| β25 | Myalgia and unspecific MSK pain (Symptoms [yes] | 1,17 | 0,1451 | |
| β26 | Generalized pain [yes] | 1,141 | 0,1239 | |
| β27 | Fever [yes] | 1,242 | 0,1946 | |
| β28 | Weight loss and night sweats [yes] | 1,189 | 0,1589 | |
| β29 | Skin conditions [yes] | 1,13 | 0,1152 | |
| β30 | fatigue and decreased general condition [yes] | 1,144 | 0,1255 | |
| β31 | Dyspnoea [yes] | 1,154 | 0,1333 | |
| β32 | abdominal complaints [yes] | 1,13 | 0,1152 | |
| β33 | ENT complaints [yes] | 1,11 | 0,09906 | |
| | | | | |
| Area under the ROC curve | | | | |
| Area | 0,8007 | | | |
| Std. Error | 0,02396 | | | |
| 95% confidence interval | 0,7537 to 0,8476 | | | |
| P value | <0,0001 | | | |
| | | | | |
| Classification table | Predicted 0 | Predicted 1 | Total | % Correctly classified |
| Observed 0 | 75 | 54 | 129 | 58,14 |
| Observed 1 | 36 | 175 | 211 | 82,94 |
| Total | 111 | 229 | 340 | 73,53 |
| | | | | |
| Negative predictive power (%) | 67,57 | | | |
| Positive predictive power (%) | 76,42 | | | |
| | | | | |
| Classification cutoff | 0,5 | | | |
| | | | | |
| | | | | |
| Data summary | | | | |
| Rows in table | 340 | | | |
| Rows skipped (missing data) | 0 | | | |
| Rows analyzed (#observations) | 340 | | | |
| Number of 1 | 211 | | | |
| Number of 0 | 129 | | | |
| Number of parameter estimates | 34 | | | |
| #observations/#parameters | 10 | | | |
| # of 1/#parameters | 6,2 | | | |
| # of 0/#parameters | 3,8 | | | |
| Number of parameter estimates | 35 | | | |
| #observations/#parameters | 9,7 | | | |
| # of 1/#parameters | 6 | | | |
| # of 0/#parameters | 3,7 | | | |
Supplementary Table ST3. Logistic regression model without rheumatological consultation (RC)
Detailed outcome of our logistic regression model for predicting an inflammatory rheumatic disease (IRD) diagnosis at discharge without RC. *** p<0.001, ** p<0.01, * p<0.05.

## Slide 4
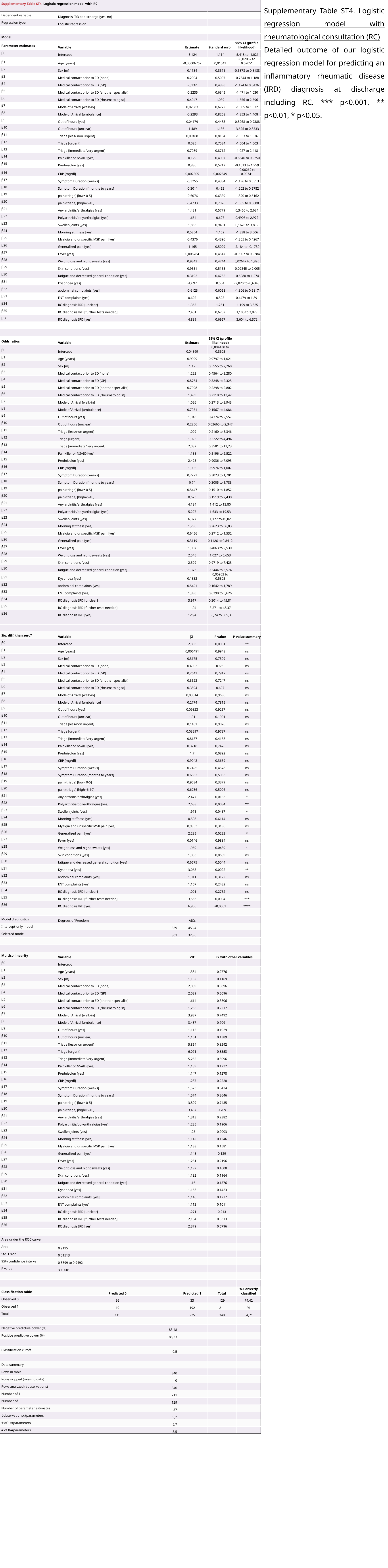

| Supplementary Table ST4. Logistic regression model with RC | | | | | |
| --- | --- | --- | --- | --- | --- |
| Dependent variable | Diagnosis IRD at discharge [yes, no] | | | | |
| Regression type | Logistic regression | | | | |
| | | | | | |
| Model | | | | | |
| Parameter estimates | Variable | Estimate | Standard error | 95% CI (profile likelihood) | 95% CI (profile likelihood) |
| β0 | Intercept | -3,124 | 1,114 | -5,418 to -1,021 | -5,418 to -1,021 |
| β1 | Age [years] | -0,00006762 | 0,01042 | -0,02052 to 0,02051 | -0,02052 to 0,02051 |
| β2 | Sex [m] | 0,1134 | 0,3571 | -0,5878 to 0,8188 | -0,5878 to 0,8188 |
| β3 | Medical contact prior to ED [none] | 0,2004 | 0,5007 | -0,7844 to 1,188 | -0,7844 to 1,188 |
| β4 | Medical contact prior to ED [GP] | -0,132 | 0,4998 | -1,124 to 0,8436 | -1,124 to 0,8436 |
| β5 | Medical contact prior to ED [another specialist] | -0,2235 | 0,6345 | -1,471 to 1,030 | -1,471 to 1,030 |
| β6 | Medical contact prior to ED [rheumatologist] | 0,4047 | 1,039 | -1,556 to 2,596 | -1,556 to 2,596 |
| β7 | Mode of Arrival [walk-in] | 0,02583 | 0,6772 | -1,305 to 1,372 | -1,305 to 1,372 |
| β8 | Mode of Arrival [ambulance] | -0,2293 | 0,8268 | -1,853 to 1,408 | -1,853 to 1,408 |
| β9 | Out of hours [yes] | 0,04179 | 0,4483 | -0,8268 to 0,9388 | -0,8268 to 0,9388 |
| β10 | Out of hours [unclear] | -1,489 | 1,136 | -3,625 to 0,8533 | -3,625 to 0,8533 |
| β11 | Triage [less/ non urgent] | 0,09408 | 0,8104 | -1,533 to 1,676 | -1,533 to 1,676 |
| β12 | Triage [urgent] | 0,025 | 0,7584 | -1,504 to 1,503 | -1,504 to 1,503 |
| β13 | Triage [immediate/very urgent] | 0,7089 | 0,8712 | -1,027 to 2,418 | -1,027 to 2,418 |
| β14 | Painkiller or NSAID [yes] | 0,129 | 0,4007 | -0,6546 to 0,9250 | -0,6546 to 0,9250 |
| β15 | Prednisolon [yes] | 0,886 | 0,5212 | -0,1013 to 1,959 | -0,1013 to 1,959 |
| β16 | CRP [mg/dl] | 0,002305 | 0,002549 | -0,00262 to 0,00741 | -0,002622 to 0,007409 |
| β17 | Symptom Duration [weeks] | -0,3255 | 0,4384 | -1,196 to 0,5313 | -1,196 to 0,5313 |
| β18 | Symptom Duration [months to years] | -0,3011 | 0,452 | -1,202 to 0,5782 | -1,202 to 0,5782 |
| β19 | pain (triage) [low= 0-5] | -0,6076 | 0,6339 | -1,890 to 0,6162 | -1,890 to 0,6162 |
| β20 | pain (triage) [high=6-10] | -0,4733 | 0,7026 | -1,885 to 0,8880 | -1,885 to 0,8880 |
| β21 | Any arthritis/arthralgias [yes] | 1,431 | 0,5779 | 0,3450 to 2,624 | 0,3450 to 2,624 |
| β22 | Polyarthritis/polyarthralgias [yes] | 1,654 | 0,627 | 0,4905 to 2,972 | 0,4905 to 2,972 |
| β23 | Swollen joints [yes] | 1,853 | 0,9401 | 0,1628 to 3,892 | 0,1628 to 3,892 |
| β24 | Morning stiffness [yes] | 0,5854 | 1,152 | -1,338 to 3,606 | -1,338 to 3,606 |
| β25 | Myalgia and unspecific MSK pain [yes] | -0,4376 | 0,4396 | -1,305 to 0,4267 | -1,305 to 0,4267 |
| β26 | Generalized pain [yes] | -1,165 | 0,5099 | -2,184 to -0,1730 | -2,184 to -0,1730 |
| β27 | Fever [yes] | 0,006784 | 0,4647 | -0,9007 to 0,9284 | -0,9007 to 0,9284 |
| β28 | Weight loss and night sweats [yes] | 0,9343 | 0,4744 | 0,02647 to 1,895 | 0,02647 to 1,895 |
| β29 | Skin conditions [yes] | 0,9551 | 0,5155 | -0,02845 to 2,005 | -0,02845 to 2,005 |
| β30 | fatigue and decreased general condition [yes] | 0,3192 | 0,4782 | -0,6080 to 1,274 | -0,6080 to 1,274 |
| β31 | Dyspnoea [yes] | -1,697 | 0,554 | -2,820 to -0,6343 | -2,820 to -0,6343 |
| β32 | abdominal complaints [yes] | -0,6123 | 0,6058 | -1,806 to 0,5817 | -1,806 to 0,5817 |
| β33 | ENT complaints [yes] | 0,692 | 0,593 | -0,4479 to 1,891 | -0,4479 to 1,891 |
| β34 | RC diagnosis IRD [unclear] | 1,365 | 1,251 | -1,199 to 3,825 | -1,199 to 3,825 |
| β35 | RC diagnosis IRD [further tests needed] | 2,401 | 0,6752 | 1,185 to 3,879 | 1,185 to 3,879 |
| β36 | RC diagnosis IRD [yes] | 4,839 | 0,6957 | 3,604 to 6,372 | 3,604 to 6,372 |
| | | | | | |
| | | | | | |
| Odds ratios | Variable | Estimate | 95% CI (profile likelihood) | | |
| β0 | Intercept | 0,04399 | 0,004438 to 0,3603 | | |
| β1 | Age [years] | 0,9999 | 0,9797 to 1,021 | | |
| β2 | Sex [m] | 1,12 | 0,5555 to 2,268 | | |
| β3 | Medical contact prior to ED [none] | 1,222 | 0,4564 to 3,280 | | |
| β4 | Medical contact prior to ED [GP] | 0,8764 | 0,3248 to 2,325 | | |
| β5 | Medical contact prior to ED [another specialist] | 0,7998 | 0,2298 to 2,802 | | |
| β6 | Medical contact prior to ED [rheumatologist] | 1,499 | 0,2110 to 13,42 | | |
| β7 | Mode of Arrival [walk-in] | 1,026 | 0,2713 to 3,943 | | |
| β8 | Mode of Arrival [ambulance] | 0,7951 | 0,1567 to 4,086 | | |
| β9 | Out of hours [yes] | 1,043 | 0,4374 to 2,557 | | |
| β10 | Out of hours [unclear] | 0,2256 | 0,02665 to 2,347 | | |
| β11 | Triage [less/non urgent] | 1,099 | 0,2160 to 5,346 | | |
| β12 | Triage [urgent] | 1,025 | 0,2222 to 4,494 | | |
| β13 | Triage [immediate/very urgent] | 2,032 | 0,3581 to 11,23 | | |
| β14 | Painkiller or NSAID [yes] | 1,138 | 0,5196 to 2,522 | | |
| β15 | Prednisolon [yes] | 2,425 | 0,9036 to 7,093 | | |
| β16 | CRP [mg/dl] | 1,002 | 0,9974 to 1,007 | | |
| β17 | Symptom Duration [weeks] | 0,7222 | 0,3023 to 1,701 | | |
| β18 | Symptom Duration [months to years] | 0,74 | 0,3005 to 1,783 | | |
| β19 | pain (triage) [low= 0-5] | 0,5447 | 0,1510 to 1,852 | | |
| β20 | pain (triage) [high=6-10] | 0,623 | 0,1519 to 2,430 | | |
| β21 | Any arthritis/arthralgias [yes] | 4,184 | 1,412 to 13,80 | | |
| β22 | Polyarthritis/polyarthralgias [yes] | 5,227 | 1,633 to 19,53 | | |
| β23 | Swollen joints [yes] | 6,377 | 1,177 to 49,02 | | |
| β24 | Morning stiffness [yes] | 1,796 | 0,2623 to 36,83 | | |
| β25 | Myalgia and unspecific MSK pain [yes] | 0,6456 | 0,2712 to 1,532 | | |
| β26 | Generalized pain [yes] | 0,3119 | 0,1126 to 0,8412 | | |
| β27 | Fever [yes] | 1,007 | 0,4063 to 2,530 | | |
| β28 | Weight loss and night sweats [yes] | 2,545 | 1,027 to 6,653 | | |
| β29 | Skin conditions [yes] | 2,599 | 0,9719 to 7,423 | | |
| β30 | fatigue and decreased general condition [yes] | 1,376 | 0,5444 to 3,574 | | |
| β31 | Dyspnoea [yes] | 0,1832 | 0,05962 to 0,5303 | | |
| β32 | abdominal complaints [yes] | 0,5421 | 0,1642 to 1,789 | | |
| β33 | ENT complaints [yes] | 1,998 | 0,6390 to 6,626 | | |
| β34 | RC diagnosis IRD [unclear] | 3,917 | 0,3014 to 45,81 | | |
| β35 | RC diagnosis IRD [further tests needed] | 11,04 | 3,271 to 48,37 | | |
| β36 | RC diagnosis IRD [yes] | 126,4 | 36,74 to 585,3 | | |
| | | | | | |
| | | | | | |
| Sig. diff. than zero? | Variable | |Z| | P value | P value summary | P value summary |
| β0 | Intercept | 2,803 | 0,0051 | \*\* | \*\* |
| β1 | Age [years] | 0,006491 | 0,9948 | ns | ns |
| β2 | Sex [m] | 0,3175 | 0,7509 | ns | ns |
| β3 | Medical contact prior to ED [none] | 0,4002 | 0,689 | ns | ns |
| β4 | Medical contact prior to ED [GP] | 0,2641 | 0,7917 | ns | ns |
| β5 | Medical contact prior to ED [another specialist] | 0,3522 | 0,7247 | ns | ns |
| β6 | Medical contact prior to ED [rheumatologist] | 0,3894 | 0,697 | ns | ns |
| β7 | Mode of Arrival [walk-in] | 0,03814 | 0,9696 | ns | ns |
| β8 | Mode of Arrival [ambulance] | 0,2774 | 0,7815 | ns | ns |
| β9 | Out of hours [yes] | 0,09323 | 0,9257 | ns | ns |
| β10 | Out of hours [unclear] | 1,31 | 0,1901 | ns | ns |
| β11 | Triage [less/non urgent] | 0,1161 | 0,9076 | ns | ns |
| β12 | Triage [urgent] | 0,03297 | 0,9737 | ns | ns |
| β13 | Triage [immediate/very urgent] | 0,8137 | 0,4158 | ns | ns |
| β14 | Painkiller or NSAID [yes] | 0,3218 | 0,7476 | ns | ns |
| β15 | Prednisolon [yes] | 1,7 | 0,0892 | ns | ns |
| β16 | CRP [mg/dl] | 0,9042 | 0,3659 | ns | ns |
| β17 | Symptom Duration [weeks] | 0,7425 | 0,4578 | ns | ns |
| β18 | Symptom Duration [months to years] | 0,6662 | 0,5053 | ns | ns |
| β19 | pain (triage) [low= 0-5] | 0,9584 | 0,3379 | ns | ns |
| β20 | pain (triage) [high=6-10] | 0,6736 | 0,5006 | ns | ns |
| β21 | Any arthritis/arthralgias [yes] | 2,477 | 0,0133 | \* | \* |
| β22 | Polyarthritis/polyarthralgias [yes] | 2,638 | 0,0084 | \*\* | \*\* |
| β23 | Swollen joints [yes] | 1,971 | 0,0487 | \* | \* |
| β24 | Morning stiffness [yes] | 0,508 | 0,6114 | ns | ns |
| β25 | Myalgia and unspecific MSK pain [yes] | 0,9953 | 0,3196 | ns | ns |
| β26 | Generalized pain [yes] | 2,285 | 0,0223 | \* | \* |
| β27 | Fever [yes] | 0,0146 | 0,9884 | ns | ns |
| β28 | Weight loss and night sweats [yes] | 1,969 | 0,0489 | \* | \* |
| β29 | Skin conditions [yes] | 1,853 | 0,0639 | ns | ns |
| β30 | fatigue and decreased general condition [yes] | 0,6675 | 0,5044 | ns | ns |
| β31 | Dyspnoea [yes] | 3,063 | 0,0022 | \*\* | \*\* |
| β32 | abdominal complaints [yes] | 1,011 | 0,3122 | ns | ns |
| β33 | ENT complaints [yes] | 1,167 | 0,2432 | ns | ns |
| β34 | RC diagnosis IRD [unclear] | 1,091 | 0,2752 | ns | ns |
| β35 | RC diagnosis IRD [further tests needed] | 3,556 | 0,0004 | \*\*\* | \*\*\* |
| β36 | RC diagnosis IRD [yes] | 6,956 | <0,0001 | \*\*\*\* | \*\*\*\* |
| | | | | | |
| Model diagnostics | Degrees of Freedom | AICc | | | |
| Intercept-only model | 339 | 453,4 | | | |
| Selected model | 303 | 323,6 | | | |
| | | | | | |
| | | | | | |
| Multicollinearity | Variable | VIF | R2 with other variables | | |
| β0 | Intercept | | | | |
| β1 | Age [years] | 1,384 | 0,2776 | | |
| β2 | Sex [m] | 1,132 | 0,1169 | | |
| β3 | Medical contact prior to ED [none] | 2,039 | 0,5096 | | |
| β4 | Medical contact prior to ED [GP] | 2,039 | 0,5096 | | |
| β5 | Medical contact prior to ED [another specialist] | 1,614 | 0,3806 | | |
| β6 | Medical contact prior to ED [rheumatologist] | 1,285 | 0,2217 | | |
| β7 | Mode of Arrival [walk-in] | 3,987 | 0,7492 | | |
| β8 | Mode of Arrival [ambulance] | 3,437 | 0,7091 | | |
| β9 | Out of hours [yes] | 1,115 | 0,1029 | | |
| β10 | Out of hours [unclear] | 1,161 | 0,1389 | | |
| β11 | Triage [less/non urgent] | 5,854 | 0,8292 | | |
| β12 | Triage [urgent] | 6,071 | 0,8353 | | |
| β13 | Triage [immediate/very urgent] | 5,252 | 0,8096 | | |
| β14 | Painkiller or NSAID [yes] | 1,139 | 0,1222 | | |
| β15 | Prednisolon [yes] | 1,147 | 0,1278 | | |
| β16 | CRP [mg/dl] | 1,287 | 0,2228 | | |
| β17 | Symptom Duration [weeks] | 1,523 | 0,3434 | | |
| β18 | Symptom Duration [months to years] | 1,574 | 0,3646 | | |
| β19 | pain (triage) [low= 0-5] | 3,899 | 0,7435 | | |
| β20 | pain (triage) [high=6-10] | 3,437 | 0,709 | | |
| β21 | Any arthritis/arthralgias [yes] | 1,313 | 0,2382 | | |
| β22 | Polyarthritis/polyarthralgias [yes] | 1,235 | 0,1906 | | |
| β23 | Swollen joints [yes] | 1,25 | 0,2003 | | |
| β24 | Morning stiffness [yes] | 1,142 | 0,1246 | | |
| β25 | Myalgia and unspecific MSK pain [yes] | 1,188 | 0,1581 | | |
| β26 | Generalized pain [yes] | 1,148 | 0,129 | | |
| β27 | Fever [yes] | 1,281 | 0,2196 | | |
| β28 | Weight loss and night sweats [yes] | 1,192 | 0,1608 | | |
| β29 | Skin conditions [yes] | 1,132 | 0,1164 | | |
| β30 | fatigue and decreased general condition [yes] | 1,16 | 0,1376 | | |
| β31 | Dyspnoea [yes] | 1,166 | 0,1423 | | |
| β32 | abdominal complaints [yes] | 1,146 | 0,1277 | | |
| β33 | ENT complaints [yes] | 1,113 | 0,1011 | | |
| β34 | RC diagnosis IRD [unclear] | 1,271 | 0,213 | | |
| β35 | RC diagnosis IRD [further tests needed] | 2,134 | 0,5313 | | |
| β36 | RC diagnosis IRD [yes] | 2,379 | 0,5796 | | |
| | | | | | |
| Area under the ROC curve | | | | | |
| Area | 0,9195 | | | | |
| Std. Error | 0,01513 | | | | |
| 95% confidence interval | 0,8899 to 0,9492 | | | | |
| P value | <0,0001 | | | | |
| | | | | | |
| | | | | | |
| Classification table | Predicted 0 | Predicted 1 | Total | | % Correctly classified |
| Observed 0 | 96 | 33 | 129 | | 74,42 |
| Observed 1 | 19 | 192 | 211 | | 91 |
| Total | 115 | 225 | 340 | | 84,71 |
| | | | | | |
| Negative predictive power (%) | 83,48 | | | | |
| Positive predictive power (%) | 85,33 | | | | |
| | | | | | |
| Classification cutoff | 0,5 | | | | |
| | | | | | |
| Data summary | | | | | |
| Rows in table | 340 | | | | |
| Rows skipped (missing data) | 0 | | | | |
| Rows analyzed (#observations) | 340 | | | | |
| Number of 1 | 211 | | | | |
| Number of 0 | 129 | | | | |
| Number of parameter estimates | 37 | | | | |
| #observations/#parameters | 9,2 | | | | |
| # of 1/#parameters | 5,7 | | | | |
| # of 0/#parameters | 3,5 | | | | |
Supplementary Table ST4. Logistic regression model with rheumatological consultation (RC)
Detailed outcome of our logistic regression model for predicting an inflammatory rheumatic disease (IRD) diagnosis at discharge including RC. *** p<0.001, ** p<0.01, * p<0.05.

## Slide 5
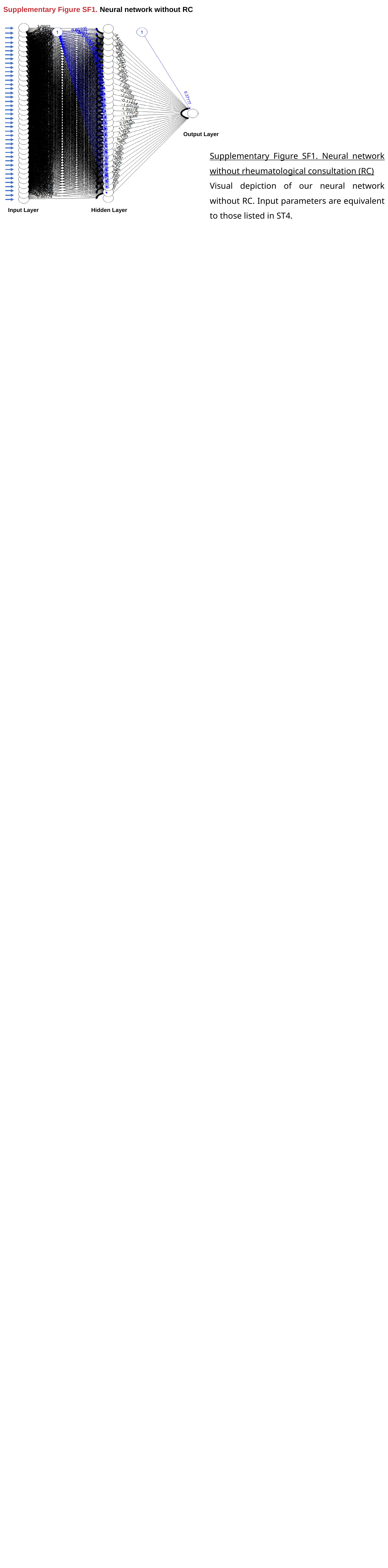

Supplementary Figure SF1. Neural network without RC
Output Layer
Supplementary Figure SF1. Neural network without rheumatological consultation (RC)
Visual depiction of our neural network without RC. Input parameters are equivalent to those listed in ST4.
Input Layer
Hidden Layer
